# Single‐cell transcriptomics in human skin research: available technologies, technical considerations and disease applications

**DOI:** 10.1111/exd.14547

**Published:** 2022-03-04

**Authors:** Georgios Theocharidis, Stavroula Tekkela, Aristidis Veves, John A. McGrath, Alexandros Onoufriadis

**Affiliations:** ^1^ Joslin‐Beth Israel Deaconess Foot Center and The Rongxiang Xu, MD, Center for Regenerative Therapeutics Beth Israel Deaconess Medical Center Harvard Medical School Boston Massachusetts USA; ^2^ St John’s Institute of Dermatology School of Basic and Medical Biosciences King’s College London London UK

**Keywords:** scRNA‐seq, single‐cell sequencing, single‐cell transcriptomics, skin research

## Abstract

Single‐cell technologies have revolutionized research in the last decade, including for skin biology. Single‐cell RNA sequencing has emerged as a powerful tool allowing the dissection of human disease pathophysiology at unprecedented resolution by assessing cell‐to‐cell variation, facilitating identification of rare cell populations and elucidating cellular heterogeneity. In dermatology, this technology has been widely applied to inflammatory skin disorders, fibrotic skin diseases, wound healing complications and cutaneous neoplasms. Here, we discuss the available technologies and technical considerations of single‐cell RNA sequencing and describe its applications to a broad spectrum of dermatological diseases.

## 1 INTRODUCTION

The human skin is defined by a multilayer architecture based on diverse cell populations of mainly keratinocytes and fibroblasts, as well as various immune cells, melanocytes, adipocytes and endothelial cells that orchestrate events leading to wound repair of pathogenic infections and exposure to ultraviolet radiation and toxic compounds.^1^ The transcriptomic profile of skin can provide information on gene expression, non‐coding regulatory elements and gene splicing, and therefore shed light on skin physiology and pathology. Earlier approaches such as bulk RNA‐seq and microarrays provided information about the average transcriptome status of an entire tissue or sample.^2^ Since the first single‐cell RNA‐seq (scRNA‐seq) study conducted by Tang et al. the development of single‐cell transcriptomics led to the revelation of gene expression variability amongst identical or distinct cell types.^3^ Importantly, scRNA‐seq allowed researchers to uncover *de novo* cell populations and lineages that may be implicated in differentiation and development.^4^ Moreover, scRNA‐seq can be used as a diagnostic tool in precision medicine by deciphering disease‐related biomarkers and pathways.^5^ This approach can be very promising in drug development, in which cell‐specific transcriptomic responses to treatment can be analysed.^6^

The aim of this review is to summarize the available technologies and the technical considerations of single‐cell transcriptomics, as well as provide a comprehensive compendium of scRNA‐seq applications in dermatological research.

## 2 AVAILABLE TECHNOLOGIES

Currently, available technologies of scRNA‐seq library generation comprise of cell lysis, reverse transcription into first‐strand cDNA, second‐strand synthesis and cDNA amplification.^7^ These technologies can be broadly classified into two categories: full‐length transcriptome sequencing and 3’ or 5’ end counting‐based sequencing.^8^ Smart‐seq is a full‐length transcription sequencing method based on a switching mechanism, in which nucleotides at the end of the RNA template are added, allowing the reverse transcriptase to synthesize the complementary cDNA strand.^9^ Smart‐seq2, which is the most widely used smart‐seq technology, provides higher sensitivity and efficiency in capturing RNA molecules.^10^ On the contrary, a popular 3’ or 5’ end counting‐based approach is the droplet microfluidics technology known as Drop‐seq,^11^ which encapsulates cells into independent microdroplets with unique barcoded beads. Each bead has a cellular barcode which is unique to each droplet, as well as unique molecular identifiers (UMIs) representing each RNA molecule.^12^, ^13^ This protocol has been developed further and commercialized with the use of the *Chromium* instrument (10× Genomics), which is widely used in dermatological research projects.^14^ The 10× technology has also developed protocols which conduct immune cell mapping and profiling of specific developing leukocytes by screening the VDJ leukocyte‐specific genes of T‐cell immune receptors (TCR).^15^ In another microfluidic technology, characterized as Seq‐well, cells are incorporated into capture beads that are confined in subnanoliter wells and sealed with a semipermeable membrane. In the membrane, the beads are removed, followed by cell lysis and mRNA capture.^16^ The first commercially available Seq‐Well microfluidic platform was the C1 by Fluidigm.^17^ Hughes et al. developed a new version of Seq‐well, Seq‐Well S,^3^ in which a randomly primed second‐strand synthesis as a second oligonucleotide handle is established after reverse transcription.^18^ This method was applied to certain dermatological diseases and is suggested to be simpler, more compatible with fragile cells and able to manage more samples in parallel.^18^ Each of the methods above has its own benefits and drawbacks. Although Smart‐seq provides a higher coverage amongst transcripts and alternatively spliced mRNA, Drop‐seq enables more cells to be sequenced simultaneously, whereas Seq‐well prevents cross‐contamination between samples.^19^ A novel scRNA‐seq technique is Smart‐seq3, which incorporates full‐length coverage and a 5’UMI tagging strategy.^20^ This protocol allows a dramatic increase of sensitivity and can estimate gene expression in a larger number of cells.^20^

## 3 TECHNICAL CONSIDERATIONS

**FIGURE 1:**
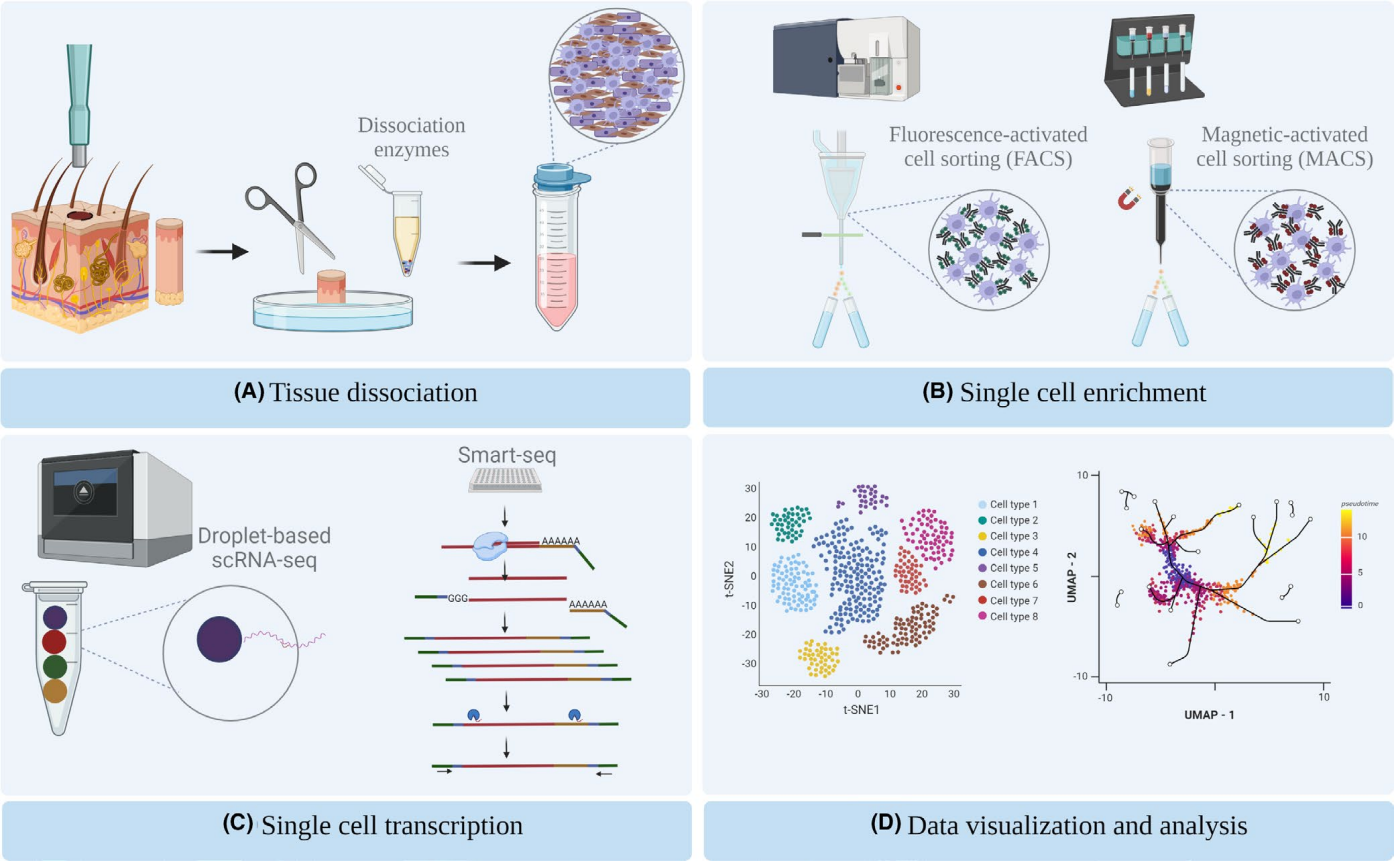
Model workflow of a scRNA‐seq experiment using skin tissue. (A) Skin punch biopsy is processed into a single‐cell solution by mechanical and enzymatical dissociation. (B) Single cells of interest are selected via FACS or MACS. (C) Genomic libraries are generated by droplet‐based or Smart‐seq technologies. (D) Visualization of scRNA‐seq data using t‐distributed stochastic neighbour embedding (tSNE) and Uniform Manifold Approximation and Projection (UMAP) showing cellular trajectories

The scRNA‐seq experimental procedure consists of four main steps: sample preparation, cell enrichment, library preparation and data analysis (Figure 1).^7^ In regard to the isolation of single cells from skin samples, punch biopsies or larger specimens are obtained and dissociated via mechanical or enzymatic treatment. Because the various layers of the skin have different cell compositions and properties, single‐cell dissociation can be quite challenging.^21^ Multiple single‐cell dissociation approaches are available depending on whether the dermis, epidermis or both are needed for each experiment. For example, a research study that aimed to resolve the basal keratinocyte transition states isolated the epidermal tissue,^22^ while in a study aiming at profiling fibroblast subpopulations, the epidermis was discarded, and the dermis was processed.^23^

Typically, whole‐skin dissociation kits such as the gentleMACS (Miltenyi Biotec) dissociation system can be used.^23^, ^24^ Nevertheless, some dissociation enzymes included in the kit favour the isolation of fibroblasts and might provoke the elimination of a subpopulation of immune cells or epidermal cells, abrogating the whole‐cell map of the sample. Alternatively, one can incubate the whole skin with dispase, leading to dermal‐epidermal dissociation.^25^ Keratinocytes, melanocytes and epidermal immune cells are further obtained by the application of trypsin and fibroblasts by digestion with collagenase.^26^ Mechanical means are also used, such as mashing, dicing or slicing, enhancing the whole process.^7^, ^26^ Tissue dissociation can vary from 2 h to overnight.^26^ During incubation, the activity of each enzyme must be taken into consideration since longer incubations can negatively impact cell viability and induce mechanical stress or trigger immune activation to the cells.^21^

An alternative approach for skin cell acquisition is the application of suction blistering, whereby an artificial blister is introduced to the skin, eliciting dermal‐epidermal dissociation and the formation of a fluid skin sample combined with interstitial fluid.^27^ The liquid nature of the biopsy can enhance single‐cell dissociation.^28^ A major limitation of this method is the inability to capture deeper dermal regions, and therefore, dermal, endothelial cell and macrophage (Mφ) information cannot be provided.^27^, ^28^

Another parameter to be considered is the possibility of samples being frozen prior to processing, allowing sample processing at different time points. However, reduction of cell numbers, and the alleviation of the cell transcriptome, are two major considerations. Several cryoprotectant agents used in tissue preservation have facilitated post‐thaw capture of an adequate number of cells, mainly fibroblasts.^29^ Another proposed method of preserving the sample is by freezing the single‐cell solution after dissociation, which has shown satisfactory cell viability and integrity.^30^ However, working with fresh tissue when needed is preferred, and multiple freeze and thaw cycles should be avoided.^21^

An unbiased view of the cellular composition of the sample and the projection of its cellular heterogeneity is revealed when one captures all desired cells. Therefore, quality check and specific cell isolation are performed in the cell enrichment step. Obtaining a pure single‐cell solution without cell debris, fragmented, or dead and apoptotic cells can reduce artifacts during downstream analysis.^31^ Quality check can be done manually by using an inverted microscope and micropipettes, or by applying negative charge in a patch pipette, a method known as micromanipulation.^7^ However, these methods are low‐throughput and very laborious. High throughput methods such as fluorescence‐activated cell sorting (FACS) or magnetic‐activated cell sorting (MACS) are predominantly used.^31^ FACS has an improved sensitivity even when a cell expresses low levels of the specific labelled markers with an antibody or with a viability dye such as DAPI.^32^ A limitation of this method is the relatively large number of cells needed as a starting material and the added stress exerted on the cells during the sorting process.^33^ On the contrary, MACS uses enzymes, antibodies and peptides conjugated to magnetic beads for sorting, requiring less time and equipment, but lacks sensitivity and specificity.^31^, ^34^ Sorting methods can also be used to isolate specific cell populations of interest, which are further analysed as a distinct sample. For example, a study outlining the vascular endothelial cell heterogeneity in human skin has used both FACS and MACS to enrich for endothelial cells,^35^ whereas another study focused on the dendritic cell signalling in psoriasis by using FACS for dendritic and Mφ cell isolation.^36^

## 4 APPLICATIONS IN SKIN RESEARCH

### 4.1 Healthy skin

Different studies have dissected the heterogeneity of dermal fibroblasts with scRNA‐seq (Table 1). Tabib et al. reported two major fibroblast subpopulations largely defined by SFRP2 and FMO1 expression, as well as five minor subsets with distinct gene expression profiles.^37^ Immunostaining and gene ontology analyses revealed different cell morphologies and suggested specialized functions, such as extracellular matrix protein localization for SFRP2+ cells and stress response for FMO1+.^37^

**TABLE 1:** Overview of scRNA‐seq studies on human skin

Study number	Condition/research focus	Skin sample type	Number of donors	Tissue dissociation method	Single‐cell enrichment	Transcriptomic platform and chemistry	Number of transcribed cells	Reference
Healthy skin								
1	Identifying major fibroblast populations	Healthy skin from dorsal mid‐forearm	6 heathy individuals	3h with whole‐skin dissociation kit (Miltenyi), followed by gentleMACS	N/A	10x Genomics	8522	Tabib et al,^37^ 2018
2	Human fibroblast subpopulations	Healthy abdominal skin	1 healthy individual	1h in dispase II, epidermis peeled off, dermis overnight in Miltenyi whole‐skin dissociation kit	FACS for CD90+	Smart‐Seq2	184	Philipeos et al,^23^ 2018
3	Skin fibroblast heterogeneity	Surplus trunk skin of female donors removed during abdominoplasty.	3 healthy female donors	2.5h with whole‐skin dissociation kit (Miltenyi), followed by gentleMACS	FACS to exclude dead cells and debris	10x Genomics	N/A	Vorstandlechner et al,^38^ 2020
4	Skin immune cells during foetal skin development	Healthy foetal skin	6 foetal surgical discards	40–60 min in collagenase IV	FACS for CD45+	10x Genomics	N/A	Xu et al,^39^ 2021
5	T lymphocytes of foetal skin	Healthy foetal trunk and adult human surgical discards	9 foetal skin samples, 9 adult skin samples	Overnight in dispase II, 90 min in liberase 3 or Overnight in collagenase P/DNase I or overnight with whole‐skin dissociation kit (Miltenyi), followed by gentleMACS	FACS to exclude dead cells and debris, CD3+	10x Genomics	1506	Reitermaier et al,^41^ 2022
6	αβγδT cells	Foetal skin	3 foetal skin samples	3h with whole‐skin dissociation kit (Miltenyi), followed by gentleMACS	FACS to exclude dead cells and debris, CD3+	10x Genomics	N/A	Reitermaier et al,^40^ 2021
7	Human melanocyte	Healthy surgical discards from adult and foetal skin	22 healthy individuals	14h in dispase II, dermal/epidermal separation, epidermis 3 min in trypsin	FACS for KIT+ melanocytes	Smart‐seq2	9719	Belote et al,^42^ 2021
8	Myeloid cells	Healthy skin from dorsal mid‐forearm	10 heathy individuals	2h with whole‐skin dissociation kit (Miltenyi), followed by gentleMACS	N/A	10x Genomics	27 869	Xue et al,^43^ 2020
9	Basal stem cell transition states	human neonatal foreskin	5 neonatal foreskin	2h in dispase, dermal‐epidermal separation, epidermis 15 min in trypsin	FACS to exclude dead cells and debris	Droplet‐enabled single‐cell RNA sequencing	17 553	Wang et al,^22^ 2020
10	Vascular endothelial cell heterogeneity in human skin	Surgical Skin tissue discards from healthy individuals	10 healthy individuals	Overnight with dispase II, dermis 40 min in collagenase type 4/DNase I	MACS for CD31+ CD45− cells and FACS for endothelial cells	10x Genomics	N/A	Li et al,^35^ 2021
11	Eosinophilic fasciitis	Healthy skin (T helper 2 cell–fibroblast niche)	3 healthy individuals	Mechanical dissociation, overnight in collagenase XI/DNase	FACS for CD45+, PDPN+, CD45, −CD31−E‐cadherin−CD235a−, viable cells	10x Genomics	N/A	Boothby et al,^95^ 2021
12	Ageing	Eyelid skin from blepharoplasty	9 human female individuals	1h in collagenase IV/dispase/trypsin cocktail	FACS to exclude dead cells and debris	10x Genomics	35 678	Zou et al,^44^ 2021
13	Ageing	Skin from a defined, Caucasian sun‐protected skin	2 young donors, 3 old donors	1h with whole‐skin dissociation kit (Miltenyi), followed by gentleMACS	N/A	10x Genomics	15 457	Solé‐Boldo et al,^45^ 2020
14	Dermal sheath cells and ageing	Skin from upper forearm	3 young and 4 old female donors	1h with whole‐skin dissociation kit (Miltenyi), followed by gentleMACS	N/A	10x Genomics	72 −048	Ahelrs et al,^96^ 2022
Inflammatory skin disorders								
15	AD and psoriasis	Skin from the lower back close to lesions	5 healthy skin samples, 4 AD skin samples, 3 psoriasis skin samples	1h with dispase II, dermal‐epidermal separation, collagenase IV overnight	FACS for CD45−and CD45+	10x Genomics	17 000	Reynolds et al,^47^ 2021
16	AD and psoriasis	Lesional and non‐lesional skin	2 AD skin samples, 2 psoriasis skin samples	Overnight with collagenase/DNase/10% FCS/RPMI/Pen Strep/L‐glutamine	FACS for CD45+, CD3−CD19	Smart‐seq2	N/A	Nakamizo et al,^36^ 2021
17	AD	Suction blisters and skin biopsies from AD patients	8 AD patients, 7 healthy individuals	Suction blisters: 10 min in trypsin Biopsies: collagenase IV (time not specified)	FACS for CD45+ and CD45−	10x Genomics	17 −160	Rojahn. et al,^27^ 2020
18	AD	Non‐lesional and lesional skin taken from the extremities of AD patients	9 patients with AD, 7 healthy individuals	Frozen tissue cryopreserved in FBS + 10% DMSO Dissociation in liberase 2x for 15 min, trypsin for 15 min	N/A	10x Genomics	39 042	He et al,^30^ 2020
19	AD	Lesional AD skin	3 normal human skin samples, 4 AD skin samples	40 min in collagenase	FACS for viable CD45+, CD3–	10x Genomics	N/A	Alkon et al,^97^ 2021
20	AD	Suction blisters of Caucasian patients with spontaneous remission from previous moderate‐to‐severe AD	4 healed AD patients, 4 healthy individuals, 4 AD patients	N/A	FACS for CD45+	10x Genomics	14 538	Rindler et al,^28^ 2021
21	AD	Suction blisters from AD skin treated and non‐treated with dupilumab	19 AD patients	Enzymatically digested epidermis (not specified)	FACS for CD45+, CD45−	10x Genomics	47 997	Bangert et al,^6^ 2021
22	Psoriasis	Lesional psoriatic skin	3 psoriasis patients, 3 healthy individuals	2h with whole‐skin dissociation kit (Miltenyi), followed by gentleMACS	N/A	10x Genomics	24 259	Gao et al,^49^ 2021
23	Psoriasis	Epidermis of truncal skin, neonatal foreskin and psoriatic skin	9 normal skin samples (3 Scalp, 3 Truncal, 3 Neonatal), 3 Psoriatic truncal epidermis	Dermal‐epidermal separation, 2h in dispase, 15 min in Trypsin	FACS to exclude dead cells and debris	10x Genomics	92 889	Cheng et al,^50^ 2018
24	Psoriasis	Psoriatic skin	13 psoriasis patients, 5 heathy individuals	3h in dispase II, dermal/epidermal separation	N/A	10x Genomics	N/A	Kim et al,^51^ 2021
25	Psoriasis and cancer	Lesional psoriatic skin	11 patients with psoriasis, 5 healthy individuals	Overnight with Collagenase IV/DNase	FACs for CD45+CD3+CD8+	Smart‐Seq	4 575	Liu J et al,^52^ 2021
26	Langerhans cell subsets in psoriasis	Epidermis of foreskin trunk, and scalp tissue	25 psoriasis patients, 25 healthy individuals	Overnight in dispase II, dermal/epidermal separation, epidermis for 15 min in	FACS for viable CD45+HLA‐DR+CD207+	10× Genomics	3704	Liu X et al,^53^ 2021
27	Granuloma	Lesional skin from granulomas	3 granuloma patients, 3 normal skin samples	Dispase II followed by liberase	FACS to exclude dead cells and debris	10× Genomics	19 766	Wang et al,^48^ 2021
28	Granuloma	Granulomas from patients with antimicrobial responses in human leprosy	5 patients with reversal reactions, 5 patients with disseminated lepromatous leprosy	1h with dispase II, dermal/epidermal separation, epidermis 30 min in trypsin dermis 2h in collagenase II/DNAse	MACS for CD1a+	Seq‐Well S^3^	21318	Ma et al,^98^ 2021
29	Systemic Sclerosis	Dorsal mid‐forearm systemic sclerosis skin	4 systemic sclerosis patients, 4 healthy individuals	2h with whole‐skin dissociation kit (Miltenyi), followed by gentleMACS	FACS to exclude dead cells and debris	Smart‐Seq2	N/A	Apostolidis et al,^54^ 2018
30	Systemic sclerosis	Systemic sclerosis skin	12 patients with systemic sclerosis, 10 healthy individuals	2h with whole‐skin dissociation kit (Miltenyi)	N/A	10× Genomics	N/A	Tabib et al,^55^ 2021
31	Systemic Sclerosis	Lesional and non‐lesional systemic sclerosis skin	27 patients with systemic sclerosis	Enzymatically digested (not specified)	N/A	10× Genomics	N/A	Gaydosik et al,^56^ 2021
32	Systemic Sclerosis	Mid‐forearm skin	12 systemic sclerosis patients, 10 healthy individuals	2h with whole‐skin dissociation kit (Miltenyi), followed by gentleMACS	N/A	10× Genomics	65 199	Xue et al,^57^ 2021
33	Localized Scleroderma	Fresh and frozen localized sclerodermas	3 patients with localized scleroderma	Frozen tissue in cryostore 3h with whole‐skin dissociation kit (Miltenyi), followed by gentleMACS	N/A	10× Genomics	14 901	Mirizio et al,^99^ 2020
34	Cutaneous lupus erythematosus (CLE)	Lesional and sun‐protected non‐lesional CLE skin	7 patients with active CLE, 14 healthy individuals	Overnight with dispase, dermal/epidermal separation, epidermis 1h in trypsin, dermis 1.5h in collagenase	N/A	10× Genomics	46 540	Billi et al,^63^ 2021
35	Dermatomyositis and lupus erythematosus	Lesional and non‐lesional skin from a dermatomyositis, lupus erythematosus patients	2 dermatomyositis patients, 2 lupus erythematosus patients, 2 healthy individuals	Overnight in dispase, dermal‐epidermal separation, epidermis 1h in trypsin/DNase I, dermis 2h in collagenase I, V	N/A	10× Genomics	N/A	Tsoi et al,^100^ 2020
36	Lupus erythematosus	Frozen and fresh healthy skin of patients with lupus nephritis	17 patients with lupus nephritis	Frozen tissue in cryostore for 1h, dissociation in liberase for 15 min	N/A	Fluidigm C1	4019	Der et al,^65^ 2019
37	Lupus erythematosus	Non‐lesional, non–sun‐exposed skin	12 patients with lupus nephritis, 5 healthy individuals	15 min in Liberase TL	FACS for CD4+/CD14+	Fluidigm C1	N/A	Der et al,^64^ 2017
38	Erythema migrans	Erythema migrans lesions	10 erythema migrans patients	3h with whole‐skin dissociation kit (Miltenyi), followed by gentleMACS	N/A	10× Genomics	70 000	Jiang et al,^60^ 2021
39	Hidradenitis suppurativa	Axillary lesions and surgical excisions from hidradenitis suppurativa patients	3 hidradenitis suppurativa patients, 1 heathy individual	Overnight with dispase II/collagenase II cocktail	N/A	10× Genomics	33 624	Mariottoni et al,^58^ 2021
40	Hidradenitis suppurativa	Excisional skin from patients with severe hidradenitis suppurativa	9 patients with severe hidradenitis suppurativa	Overnight in dispase, dermal/epidermal separation, epidermis for 1h n trypsin/ DNase I, dermis 1.5h in collagenase II, collagenase V	N/A	10× Genomics	30 636	Gudjonsson JE et al,^59^ 2020
41	Human vitiligo	Suction blisters of lesional and non‐lesional human vitiligo skin	10 individuals with active vitiligo, 7 heathy individuals	Suction blisters	N/A	Drop‐seq	32 405	Gellatly et al,^66^ 2021
42	Human Papillomavirus infection	Warts from the chest and elbow and normal skin	1 immunosuppressed patient	Overnight in dispase, epidermis peeled off, epidermis 2 min in trypsin	FACS to exclude dead cells and debris	10× Genomics	15 105	Devitt et al,^62^ 2021
43	Psoriasis, eczema, AD, erythrokeratodermia variabilis	Epidermis from lesional skin from patients with psoriasis, AD, erythrokeratodermia variabilis	3 psoriasis patients, 1 AD patient, 1 Erythrokeratodermia variabilis patient, 3 healthy individuals	2h in dispase, dermal‐epidermal separation, 15 min in trypsin	FACS to exclude dead cells and debris	10× Genomics	59 502	Harirchian et al,^46^ 2019
44	Acne, alopecia areata, granuloma annulare (GA), leprosy, and psoriasis	Skin from acne, alopecia areata, granuloma annulare, leprosy, and psoriasis patients	4 patients with acnes, 1 patient with alopecia, 2 patients with granuloma annulare, 4 patients with leprosy, 5 patients with psoriasis, 3 normal skin samples	1h with dispase II, dermal/epidermal separation, Epidermis: 30 min in trypsin/ DNAse Dermis: 2h in collagenase II/DNase	MACS for CD1A+	Seq‐Well S^3^	38 274	Huges et al,^18^ 2020
45	Drug‐induced hypersensitivity syndrome/drug reaction with eosinophilia and systemic symptoms (DiHS/DRESS)	Lesional skin of patient with refractory DiHS/DRESS	1 patient with refractory DiHS/DRESS, 5 healthy individuals	3h with whole‐skin dissociation kit (Miltenyi), followed by gentleMACS	FACS to exclude dead cells and debris	10× Genomics	18 218	Kim et al,^24^ 2020
Fibrotic skin diseases and impaired wound healing								
46	Keloids	Keloids	4 patients with keloids	2h with whole‐skin dissociation kit (Miltenyi), followed by gentleMACS	N/A	10× Genomics	28 064	Liu. X et al,^68^ 2021
47	Keloids	Mature keloids and normal scars	3 patients with keloids, 3 healthy individuals	2h with dispase II, dermal‐epidermal separation, dermis 2h in collagenase IV	N/A	10× Genomics	40 655	Deng et al,^67^ 2021
48	Keloids	Keloids and normal skin	2 patients with keloids, 3 patients with normal skin	Keloids: 60 min in liberase Normal skin: 1h with whole‐skin dissociation kit (Miltenyi), followed by gentleMACS	LUNA‐FL dual fluorescence cell counter to exclude dead cells and debris	10× Genomics	35 424	Shim et al,^101^ 2022
49	Pressure ulcers	Skin from the excision site of patients with ulcers	5 patients with spinal cord injury with grade IV pressure ulcers, 4 healthy individuals	Overnight with dispase, dermal/epidermal separation, epidermis 10 minutes in trypsin	N/A	Smart‐seq2	1170	Li et al,^71^ 2021
50	Diabetic foot ulcers	Foot and forearm skin; healed and non‐healed ulcers of patients with Diabetes Mellitus	10 non‐diabetic patients, 10 diabetic patients with no foot ulceration, 11 diabetic patients with foot ulcers, 4 non‐diabetic patients with arm biopsy, 2 diabetic patients with no foot ulcers giving arm biopsies, 5 diabetic patients with foot ulcers giving arm biopsies	Overnight with dispase II, 90 min in Collagenase P	N/A	10× Genomics	94 325	Theocharidis et al,^70^ 2022
51	Diabetic foot ulcers	Ulcers and foot skin from healthy and diabetic patients	4 diabetic patients’ ulcers, 4 diabetic patients’ foot skin, 4 healthy individuals	2h in collagenase P/dispase II/ DNase I cocktail	N/A	10× Genomics	9878	Theocharidis et al,^69^ 2020
52	Hypertrophic scars	Skin from hypertrophic scars	3 resected scar tissue, 3 healthy skin samples	2.5h with whole‐skin dissociation kit (Miltenyi)	FACS to exclude dead cells and debris	10× Genomics	N/A	Vorstandlechner et al,^38^ 2021
Cutaneous neoplasms								
53	Cutaneous B‐cell and T‐cell lymphoma	Skin from a brownish plaque on the left flank and purple tumor on the left abdomen	1 cutaneous follicle centre lymphoma patient	2h with whole‐skin dissociation kit (Miltenyi), followed by gentleMACS	N/A	10× Genomics	8654	Joniak et al,^77^ 2021
54	Cutaneous T‐cell lymphoma	Lesional skin from advance stage Cutaneous T‐cell lymphoma patients	5 patents with Cutaneous T‐cell lymphoma, 5 healthy individuals	2h with whole‐skin dissociation kit (Miltenyi), followed by gentleMACS	N/A	10× Genomics	14 119	Gaydosik et al,^78^ 2019
55	Cutaneous T‐cell lymphoma (mycosis fungoides)	Lesional and non‐lesional skin from flat skin and plague/tumor lesions of mycosis fungoides patients	11 mycosis fungoides patients, 3 healthy individuals	1h with whole‐skin dissociation kit (Miltenyi), followed by gentleMACS	N/A	10× Genomics	47 172	Rindler et al,^75^ 2021
56	Mycosis fungoides	MF lesion	1 mycosis fungoides patient	30 min in collagenase IV	FACS for viable CD45+CD3+CD4+ T helper cells, other CD45+ and CD45−cells	10× Genomics	4512	Rindler et al,^76^ 2021
57	Squamous cell carcinoma (SCC)	SCC tumor	10 SCC patients	30 min in trypsin, frozen 10%DMSO/ DNase I SCC−13 media/30 min in collagenase I	FACS for CD45+	10× Genomics	50 009	Ji et al,^29^ 2020
58	Basal cell carcinoma (BCC)	BCC tumor	4 BCC patients	Overnight in dispase II/ collagenase IV cocktail, 15 min in trypsin	N/A	10× Genomics	N/A	Guerrero‐Juarez et al,^73^ 2021
59	Basal cell carcinoma (BCC)	BCC tumor	4 BCC patients	1h in collagenase, 15 min in trypsin	FACS for ItgA6+	10× Genomics	N/A	Yao et al,^72^ 2020
60	Melanoma	Melanomas	31 melanoma patients	10 min in collagenase P/DNase I	FACS for viable CD45+ or CD45	Smart‐Seq2, 10× Genomics	2987	Jerby‐Arnon et al,^80^ 2018
61	Melanoma	Melanomas with lymphoid tissue metastasis	10 melanoma patients	10 min in collagenase P, /DNase I	FACS to exclude dead cells and debris	Smart‐Seq2	4645	Tirosh et al,^79^ 2016
62	Langerhans cell histiocytosis (LCH)	LCH lesional skin	4 patients with multisystem disease, 3 patients with single‐system disease	Collagenase IV/ dispase II cocktail	FACS for CD45 CD1a CD207 viable cells	10× Genomics	N/A	Halbritter et al,^82^ 2019
63	Paget's disease	Epidermal cells of Paget's Disease skin	1 extra‐Mammary Paget's Disease patient	2h with whole‐skin dissociation kit (Miltenyi), followed by gentleMACS	FACS to exclude dead cells and debris	10× Genomics	23 511	Song et al,^81^ 2020
64	Cutaneous neurofibroma	Cutaneous neurofibromas at the globular stage	3 cutaneous neurofibroma samples	22 h with whole‐skin dissociation kit (Miltenyi)	N/A	10× Genomics	17 132	Brosseau et al,^74^ 2021

N/A, not available.

Moreover, Philippeos et al. used flow sorting to capture CD90+ mesenchymal cells from human dermis, which clustered into five subpopulations after sequencing.^23^ These included CD39+CD26− papillary dermis fibroblasts, characterized by the expression of specific minor collagens such as COL6A5 and COL23A1, CD36+ cells located in the lower dermis and representing preadipocytes and three additional groups representing cells throughout the reticular dermis.^23^ According to another publication, skin fibroblasts should be classified not only based on their anatomical location but also their transcriptome. The six fibroblast clusters which were identified did not overlay with previously established markers of papillary and reticular cells and showed inter‐cluster similarities at the transcriptional level.^38^ In addition, they were predicted to perform specific functional roles like regulation of TNF‐α or p38/MAPK signalling and the DPP4+ cell population was shown as a key producer of extracellular matrix genes, suggesting it could be a therapeutic target in fibrotic diseases.^38^

Considerable efforts have been made in recent years to characterize different cell types in embryonic and foetal human skin. To unravel the developmental dynamics of immune cells in human skin, CD45+ hematopoietic cells from foetal skin specimens at distinct gestational points were profiled with scRNA‐seq. Mφ origins and transcriptional changes were explored, establishing yolk sac‐ or hematopoietic stem cell‐derived populations.^39^ DCs, innate lymphoid, natural killer and T cells were also described in detail. The second trimester was identified as a critical time point where most of skin immune cells differentiate to a mature state, a process accompanied by metabolic reprogramming and involvement of cell type‐specific transcription factors.^39^ Two manuscripts by Reitermeier et al. characterized foetal skin T lymphocytes, revealing a previously undescribed double‐positive αβγδ functional T‐cell subset with a potential role in protecting from intrauterine infections.^40^, ^41^ By integrating scRNA‐seq with flow cytometry, *in situ* immunofluorescence and TCR repertoire profiling, they provided a comprehensive atlas of T cells during development.^41^ Finally, Belote et al. performed targeted scRNA‐seq to comprehensively characterize melanocytes across different anatomical sites, skin tones, sexes and developmental stages and defined transcriptional programs and gene expression signatures that could be applied in melanoma prognosis.^42^

A transcriptomic map of Mφ and dendritic cells (DCs) was built by performing unbiased scRNA‐seq on healthy human skin. Three Mφ and six DC populations were described, including an unreported LAMP3+ mature DC and a proliferating progenitor DC subpopulation, as well as a TREM2+ Mφ subpopulation resembling Mφ present in neurodegenerative diseases.^43^ Wang et al. defined the heterogeneity of the epidermal compartment in healthy human skin, reporting at least four basal stem cell populations in neonatal interfollicular epidermis.^22^ These cells with distinct localization were characterized by specific marker gene expression, such as proto‐oncogene PTTG1 and epigenetic modifiers HELLS or UHRF1, and were shown to have different roles in homeostasis including terminal differentiation and proliferation.^22^ Cutaneous vascular endothelial cells were recently surveyed using scRNA‐seq, identifying five major subtypes and specifying IGFBP3 and RBP7 as arteriole and SELE and MT2A as venous markers, respectively.^35^ Postcapillary and capillary endothelial cells demonstrated enhanced inflammatory‐associated gene expression pointing to an immunomodulatory role of the dermal vasculature.^35^

Analysing eyelid skin from healthy subjects across distinct ages, Zou et al. resolved the cellular composition of human skin in young, middle‐aged, and older individuals and defined molecular alterations associated with ageing.^44^ Epidermal basal cells were classified into six proliferating or quiescent subsets. Augmented chronic inflammation and attenuated basal cell self‐renewal pathways were prominent characteristics of aged skin. Fibroblasts exhibited the highest level of transcriptional variability out of all cell types during ageing and growth‐controlling transcription factor HES1 was an important driver of senescence in fibroblasts.^44^ An additional study aiming to delineate age‐related effects on fibroblasts, collected sun‐protected tissue from young and old male subjects for scRNA‐seq analysis.^45^ Four major fibroblast populations were described and categorized as secretory‐reticular, secretory‐papillary, pro‐inflammatory and mesenchymal according to their anatomical location and predicted functional role, but these identities were blurred in aged cells. Notably, aged fibroblasts also displayed a decreased number of interactions with other cell types.^45^

### 4.2 Inflammatory skin disorders

Atopic dermatitis (AD) has been one of the most extensively studied cutaneous diseases with scRNA‐seq. Harirchian and colleagues analysed epidermal cells to demonstrate that IL‐17A induced targets of A20, an NFκB inhibitor and contributor to different skin rashes, share a similar overexpression in keratinocytes not only from AD, but also from psoriasis and erythrokeratodermia variabilis.^46^ This highlights the role of keratinocytes in inflammatory skin disorders and suggests A20 skin upregulation as potential treatment.^46^ An AD study combining a suction blistering technique, which allows simultaneous proteomic profiling of interstitial fluid, alongside traditional biopsies, showed enrichment of myeloid cells and upregulated proteins of DC or Mφ origin in AD samples compared to controls.^27^ In addition, He et al. profiled both lesional and non‐lesional AD specimens and reported a novel COL6A5+ COL18A1+ lesional fibroblast subpopulation expressing CCL2 and CCL19 cytokines.^30^ The presence of a unique lesional DC population enriched for the CCL19 receptor CCR7 underscored a potential important role of fibroblast and immune cell communication.^30^

A recent study integrating flow cytometry and scRNA‐seq with published skin data sets examined the Mφ and DC landscape in AD and psoriasis and identified IL‐1B and IL‐23 producing CD14+ DC3s as potential inflammatory modulators in psoriasis.^36^ Furthermore, in an effort to characterize tissue‐resident immune memory in AD patients treated with IL‐4Rα blocker dupilumab, Bangert et al. employed scRNA‐seq and proteomics to discover persisting immune cell populations after a year of clinical remission.^6^ These included LAMP3+ CCL22+ mature DCs, CRTH2+ CD161+ T helper cells and CRTAM + cytotoxic T cells with a cytokine receptor repertoire suggestive of an epidermal alarmin cross‐talk.^6^ In a seminal publication from the Haniffa laboratory, more than half a million single cells from developing and adult healthy skin, as well as psoriatic and AD skin were profiled, to establish a skin atlas with unique cell populations enriched in disease such as F13A1+ Mφ, migratory DCs and a subset of vascular endothelial cells expressing inflammatory cytokines and leukocyte adhesion molecules.^47^ The gene signatures of these Mφ and endothelial cells bore striking similarities to their foetal counterparts, indicating a re‐emergence of developing cell states in AD and psoriasis pathogenesis and offering new insights for targeted therapeutic interventions.^47^A new single‐cell sequencing method, called second‐strand synthesis, was recently developed for enhanced gene detection and transcript capture and its efficiency was demonstrated in samples from five different inflammatory skin conditions: acne, alopecia areata, granuloma annulare (GA), leprosy and psoriasis.^18^ Findings include an overrepresentation of Tregs and an IRF4+ DC population in psoriasis, enrichment of proliferating endothelial cells in acne and immature cytotoxic T‐cell clusters in leprosy and GA, together with unique fibroblast and Mφ populations.^18^ GA immunopathogenesis was also recently investigated and CD4+ T‐cell‐derived IFN‐γ and IL‐21, as well as Mφ secreted oncostatin M, were found to be elevated in GA.^48^ As all these cytokines are involved in the JAK‐STAT signalling pathway, the authors postulated that JAK inhibitor treatment could be an effective therapeutic strategy for GA and proceeded to demonstrate improvement in five patients after treatment with JAK1/3 inhibitor tofacitinib.^48^

Numerous scRNA‐seq studies have focused on characterizing psoriatic skin. Gao et al. uncovered an immunoregulatory role of skin resident epidermal and mesenchymal cells, which express major histocompatibility complex genes and can activate DCs via secretion of LIF, IL‐6, IL‐17B, IL‐36 and CD58 cytokines to contribute to disease progression.^49^ Other reports only analysed the epidermal component and discovered a CD1C+CD301A+ myeloid DC population,^50^ or used a technique to capture emigrating immune cells from skin biopsies in order to increase the number of sequenced leukocytes without harsh enzymatic digestion, sorting or activation of characteristically plastic cell populations.^51^ This methodology revealed four distinct T17 cell subsets with a uniquely enriched IL‐17F+ IL‐1− population and a subset of semimature DCs expressing IL‐23A and IL‐36G in psoriasis.^51^ Liu and colleagues comprehensively charted the highly heterogeneous CD8+ T‐cell populations in psoriatic lesions and highlighted the increased expression of CXCL13 amongst T17 cell subsets, showing it could function as a biomarker of disease severity with a comparable or greater accuracy than IL‐17A.^52^ Finally, in a scRNA‐seq study characterizing Langerhans cells, two steady‐state (LC1 and LC2) and two activated subsets were revealed. LC2, which were more likely to be activated, bore similarities to monocytes, expressed immunosuppressive genes and were more abundant in psoriatic lesions.^53^

The Lafyatis laboratory used scRNA‐seq to gain insights into vasculopathy of systemic sclerosis (SSc) by characterizing cutaneous endothelial cells and revealed genes *APLNR* and *HSPG2*, which are mediators of Apelin/Elabela‐APLNR and TGF‐β signalling and could potentially serve as biomarkers of pathogenesis.^54^ The authors further explored the disease by analysing myofibroblast populations, which are the driver cell type of fibrosis, the most prominent manifestation of SSc on the skin.^55^ They showed that SSc myofibroblasts arise from a SFRP2^hi^ DPP4+ progenitor fibroblast population in two steps: an initial global shift of SFRP2^hi^ WIF1+ to SFRP2^hi^ PRSS23+ WIF1− fibroblasts, only a subset of which subsequently differentiate into myofibroblasts also expressing SFRP4 and FNDC1.^55^ Additional reports focused on mapping either T lymphocyte heterogeneity in SSc skin, revealing a unique CXCL13+ T‐cell subpopulation possibly promoting B‐cell responses and autoantibodies production;^56^ or myeloid cells, identifying enriched FCGR3A+ Mφ and FCN1+ monocyte‐derived DC subsets associated with severe skin SSc.^57^

In axillary lesions from hidradenitis suppurativa (HS) patients, a chronic inflammatory follicular occlusion condition, monocytes and Mφ exhibited similar transcriptomic profiles to diabetic foot ulcer cells.^58^ They also overexpressed a series of markers associated with Fc signalling, metabolic activity, type I and II interferon stimulation and were more polarized towards the M1 phenotype.^58^ In addition, excisional samples from patients with severe HS analysed with scRNA‐seq and proteomics, unmasked activation of the immune complement system together with B‐cell and plasma cell as crucial pathways contributing to HS pathogenesis.^59^

ScRNA‐seq has also been proven effective in mapping the immune response of bacterial infection lesions such as erythema migrans and leprosy granulomas, or human papillomavirus (HPV) positive lesions. Samples of erythema migrans, a skin rash and initial sign of Lyme disease, were analysed with scRNA and B‐cell receptor sequencing. Increased numbers of B cells with MHC class II upregulated genes, as well as memory B cells with IgM receptors, were found pointing to local antibody production at the skin infection site.^60^ By combining scRNA‐seq with spatial transcriptomics, Ma et al. constructed the cellular network of the antimicrobial response in leprosy granulomas.^61^ Mφ were predominantly located at the centre of the granuloma and were surrounded by lymphocytes and distinct fibroblast subpopulations. Successful antimicrobial response was regulated by IFN‐γ and IL‐1β and was orchestrated not only by immune cells but also keratinocytes, fibroblasts and endothelial cells.^61^ Incorporation of common epithelial HPV genotypes with their human counterparts during the mapping step, along with scRNA‐seq in warts of an immunosuppressed patient, allowed the detection of the alpha papillomavirus HPV78 in basal and suprabasal keratinocytes and in hair follicle stem cells and could be applied for identifying HPV transcripts with malignancy potential in specific cells.^62^

The cellular composition and molecular drivers in cutaneous lupus erythematosus lesional and non‐lesional skin were reported by Billi et al.^63^ Normal appearing skin in lupus patients was revealed as a highly type I interferon enriched environment that universally affects the gene expression of all skin cell types, while in lesional skin, accumulated CD16+ DCs arose as potent disease contributors.^63^ In other studies, focused on lupus nephritis, skin scRNA‐seq was leveraged to determine whether skin biopsies could be utilized as renal disease biomarkers.^64^ IFN‐inducible genes, including *IFI6*, *STAT1* and *IFITM1*, were indeed upregulated in keratinocytes of patients with lupus nephritis indicating a systemic response to IFN.^64^ Expanding on their previous report, the authors processed more samples and included paired renal and skin biopsies from the same individuals to confirm augmented expression of type I interferon response pathway genes in lupus patients.^65^ They also stratified patients as responders and non‐responders to treatment and found that non‐responders’ tubular epithelial cells and keratinocytes overexpressed fibrosis‐associated extracellular matrix genes.^65^

To better understand the initiation and progression of vitiligo, Gellatly et al. employed suction blistering and scRNA‐seq on affected and unaffected skin in vitiligo patients along with healthy individuals’ skin.^66^ They demonstrated the inability of lesional regulatory T cells (Tregs) to suppress autoreactive CD8+ T‐cell‐mediated depigmentation as they effectively do in non‐lesional skin and also identified the CCR5/CCL5 axis as pivotal for the cross‐talk between effector CD8+ T cells and Tregs. In both animal model and patient samples, the chemokine receptor CCR5 appeared to influence Treg function by promoting their proximity to CD8+ T cells to suppress them.^66^

### 4.3 Fibrotic skin diseases and impaired wound healing

Dysregulated cutaneous wound repair can lead to the development of keloids, abnormal fibroproliferative growths with excessive accumulation of collagen and other extracellular matrix components. To gain insights into keloid pathogenesis and aetiology, Deng et al. compared dissociated dermis of normal scar tissue with that of keloids using scRNA‐seq.^67^ They found four major fibroblast subpopulations, of which the mesenchymal group was significantly more abundant in keloids. The fibroblasts in this group overexpressed osteogenesis and chondrogenesis‐related secretory proteins, POSTN and COL11A1, and were involved in collagen overproduction.^67^ Furthermore, in another comparative investigation, keloid lesional skin was analysed together with adjacent normal tissue and different signalling pathways were identified as important disease mediators in fibroblasts and vascular endothelial cells.^68^ TGF‐β signalling molecules SMAD3 and TWIST1 were reported as upregulated in fibroblasts, while the Ephrin‐Eph pathway was activated in both fibroblasts and endothelial cells. Notably, the pathway for negative regulation of transcription of PTEN, one of the most commonly mutated tumor suppressor genes, was activated, indicating cell growth pathway overlap between keloids and some cancers.^68^ Vorstandlechner et al. studied mature hypertrophic scars from resection surgeries and discovered that serine proteases DPP4 and PLAU could potentially be implicated in scar formation.^38^ Pharmacologic inhibition or knockdown of either gene prevented TGF‐β induced fibroblast to myofibroblast differentiation and protease inhibitor BC‐11 or Sitagliptin treatment led to better collagen alignment in mouse scars.^38^

Chronic wounds, such as diabetic foot ulcers and pressure sores, are on the opposite end of the healing spectrum and characterized by failure to progress to an orderly and timely course of repair. ScRNA‐seq analysis of diabetic foot ulcers, diabetic non‐ulcerated and healthy foot skin revealed multiple fibroblast subpopulations and the ones derived from diabetic skin exhibited an injury‐associated gene expression profile suggesting that prolonged exposure to stressors such as inflammation and hyperglycaemia impacts the cells even before the development of a wound.^69^ IL‐13 and IFN‐γ expression were inhibited in ulcers and both molecules were predicted as upstream regulators in multiple cell types, which could be translated therapeutically by targeted activation for improved healing.^69^ A substantial increase of the sample size in a subsequent report allowed the comprehensive mapping of the diabetic wound healing ecosystem and comparison between patients who healed and those who did not heal their ulcers.^70^ A subtype of fibroblasts that was uniquely present in the wounds of healers was identified and corroborated with spatial transcriptomics and immunostaining. These cells were enriched for extracellular matrix and inflammation‐associated genes, including *CHI3L1*, *MMP1*, *MMP3* and *IL*‐*6*. In addition, healing ulcers also contained elevated numbers of classically activated or M1 Mφs, highlighting the importance of mounting an acute inflammatory response to successfully heal and suggesting potential interplay between healing fibroblasts and Mφs.^70^ Moreover, Li et al. profiled the transcriptome of epidermal cells from pressure ulcers, acute wounds and uninjured skin and detected increased numbers of Major Histocompatibility Complex II expressing keratinocytes in ulcers of patients with worse healing outcomes.^71^ IFN‐γ was suggested as a causative factor triggering these cells, which could also influence T‐cell activation.^71^

### 4.4 Cutaneous neoplasms

ScRNA‐seq has been an invaluable tool in various skin cancers for deconstructing complex tumor cellular heterogeneity. Basal cell carcinoma (BCC), the most common type of skin cancer, was analysed by Yao et al., discovering three prognostic cell surface markers (LYPD3, TACSTD2, LY6D) that correlate with resistance to smoothened inhibitor treatment.^72^ The AP‐1 signalling pathway was identified as a potential candidate for improved combinatorial therapies.^72^ Guerrero‐Juarez et al. described the single‐cell transcriptional states of different primary BCC subtypes and also included peri‐tumor normal skin in their analyses to define normal and malignant cells.^73^ WNT5A+ fibroblasts were identified as drivers of stromal inflammation and heat shock protein upregulation was reported as a protection mechanism in response to this inflammation to sustain tumor progression. Inhibition of heat shock proteins as the authors demonstrated with HSP70, could therefore be effective in suppressing BCC growth.^73^

Combining complementary modalities spatial transcriptomics and multiplexed ion beam imaging with scRNA‐seq, Ji et al. defined the ecosystem of squamous cell carcinoma.^29^ They discovered a tumor‐specific keratinocyte population overexpressing cellular motility, extracellular matrix disassembly and epithelial‐mesenchymal transition genes. These cells localized at the tumor leading edge surrounded by a fibrovascular niche and were important mediators of intercellular communication including cancer‐associated fibroblasts and endothelial cells. Several cell types were also revealed to be involved in immunosuppressive mechanisms, including DC inhibition, Treg recruitment and T‐cell exhaustion.^29^

Cutaneous neurofibromas are benign peripheral nerve tumors and a clinical presentation of the genetic syndrome neurofibromatosis type 1. ScRNA‐seq was employed to profile the matrisome gene expression of the tumor microenvironment cells, revealing the absence of collagen I myofibroblasts and elevated expression of collagen VI by fibroblasts instead.^74^

Rindler et al. discerned disease progression in primary cutaneous T‐cell lymphoma by performing scRNA‐seq αβ and T‐cell receptor sequencing on skin samples from patients with different stages of mycosis fungoides (MF), the most frequent type of this malignancy.^75^ Lesion progression correlated with downregulation of tissue‐resident memory T‐cell markers CXCR4 and CD69, heat shock protein HSPA1A, immunoregulatory molecules ZFP36 and TXNIP, and interleukin receptor IL7R.^75^ Furthermore, because malignant cells can spread from the skin in later stages, the authors profiled with scRNA‐seq and simultaneous V‐D‐J sequencing the tumor microenvironment of skin, blood and lymph node in a patient with advanced MF.^76^ They found skin tissue‐resident memory T cells that could switch to a more central memory‐like phenotype in circulation and could explain their migratory behaviour.^76^ A single patient's samples with concurrent MF and primary cutaneous follicle centre lymphoma (PCFCL)—the most common cutaneous B‐cell lymphoma—appearing in separate lesions were analysed with scRNA‐seq and combined T‐cell and B‐cell receptor sequencing.^77^ Two co‐occurring clonal malignancies were unveiled, with the T‐cell clone expressing Th2‐related markers while the PCFCL lesions exhibited a more Th1 skewed gene expression profile and this was reflected in the tumors micromilieu.^77^ Finally, Gayodosik and colleagues employed scRNA‐seq on lymphocytes purified from skin biopsies of advanced stage cutaneous T‐cell lymphoma patients and confirmed a large inter‐ and intratumor T‐cell gene expression heterogeneity.^78^ Patient‐specific enriched cell subpopulations and markers were outlined, demonstrating the efficiency of scRNA‐seq as a diagnostic and informational tool for personalized medicine. Additionally, a T‐cell population with a common proliferating and resistance to apoptosis gene expression signature was described.^78^

The first study to harness the technology of scRNA‐seq in human skin samples examined metastatic melanoma from 19 tumors with diverse clinical and therapeutic backgrounds.^79^ Tirosh et al. observed drug‐resistant malignant cell subpopulations that existed before treatment and were further enriched as a result of MAP kinase‐targeted treatment, a finding systematically validated in a number of melanoma cell lines.^79^ Cell‐cell interactions were inferred by deconvolution of bulk RNA‐seq melanoma profiles and a notable set of genes highly correlating with T‐cell infiltration was particularly upregulated in cancer‐associated fibroblasts. T lymphocyte diversity was also profiled and potential biomarkers to separate cytotoxic and exhausted T cells were suggested.^79^ Furthermore, Jerby‐Amon et al. sought to decipher immune checkpoint inhibitor (ICI) resistance in melanoma and established a prognostic program that could inform therapeutic approaches.^80^ Anti‐PD‐1 and anti‐CTLA‐4 treatments were predicted as effective, while CDK4/6i could reverse the resistance and resulted in improved ICI response *in vivo*. An association between immunosuppressive gene expressing Mφ and T‐cell abundance was also noted.^80^

In extramammary Paget's disease, a form of malignant intra‐epidermal adenocarcinoma characterized by the appearance of Paget cells, scRNA‐seq of one patient's epithelium provided new understanding on disease pathogenesis.^81^ KRT6C+ keratinocytes were uniquely found in diseased epidermis, while a novel cell surface marker (CD166/ALCAM) was reported in Paget cells which would allow flow sorting and more in depth study of these cells in future.^81^ Activation of mTOR signalling either through PTEN or HER2 was highlighted as an aberrant pathway in the disease and topical treatment of patients with mTOR inhibitor rapamycin effectively mitigated symptoms.^81^ Langerhans cell histiocytosis (LCH) is another relatively rare neoplasm, with lesions appearing mostly in skin and bones and predominantly affecting paediatric patients, which was recently mapped with scRNA‐seq.^82^ Two LCH subpopulations with characteristics of progenitor cells were identified and the authors posited the existence of a cell hierarchy commencing from these cells and leading to the more differentiated four states that were discovered. These four subsets expressed marker genes similarly to different immune cell types, including DCs and mature Langerhans cells, but also extracellular matrix destruction genes such as matrix metalloproteinases and aminopeptidases. The JAK/STAT signalling pathway was suggested as a master driver of the differentiation with the participation of NFKB, AP‐1, IRF8 and BATF3.^82^ Merkel cell carcinoma lesions profiled with scRNA‐seq showed a unique γδ T‐cell subpopulation with a 13‐gene pro‐inflammatory signature, which could serve as a prognostic biomarker.^83^

## 5 CONCLUSIONS AND PERSPECTIVES

Single‐cell‐based screening methods can provide a more comprehensive depiction of the multiple molecular features and gene regulation of a cell. By combining transcriptomics with proteomics and epigenomics, scientists can elucidate regulatory elements and transcription factors that affect gene expression, methylation, protein abundance and chromatin accessibility.^84^ The 10× platform enables the combination of scRNA‐seq with other complementary technologies such as ATAC‐seq, which uses a prokaryotic transposase to tag accessible regulatory regions with sequencing adaptors^85^ or CITE‐seq,^86^ which measures cell surface protein levels by using oligonucleotide‐labelled antibodies.^87^ This method can be particularly useful in better characterizing cutaneous immune cell populations that participate in multiple inflammatory signalling pathways. For instance, Liu et al. integrated scRNA‐seq with CITE‐seq data, identifying a strong correlation for transcript‐ and protein epitope‐defined APC and T cells in response to skin inflammation.^88^ Despite their high accessibility, antibody‐based approaches lack specificity and precision in protein targeting. Therefore, single‐cell proteomics technologies, which combine chromatographic and mass spectrometry methods, have been developed. One of these technologies is SCoPE‐MS, in which single cells are labelled with tandem mass tags (TMTs) and analysed together by liquid chromatography‐tandem mass spectrometry.^89^ A new high‐throughput single‐cell proteomics version has been reported, called proteoCHIP, in which cells are isolated and pooled in nanowells placed on microscopic slides. This method has identified 2000 protein groups across 158 multiplexed single cells.^90^ These proteomic technologies can provide a more precise understanding of protein structure and function, which is required in the investigation of the many structural proteins that are present in the skin.^91^.

The spatial distribution of each cell in the organism can be interrogated by a method classified as spatial transcriptomics, with the aid of fluorescent probes conducting single‐cell *in situ* hybridization or by oligonucleotide barcoding prior to sequencing.^23^ This feature can be particularly useful in the dermatologic context since the relationships between gene expression and ultrastructural tissue regions can be deconvoluted, providing information about tumor microenvironment interactions,^59^ cell fate during development and repair,^23^ and spatial composition of inflammation.^92^ For instance, spatial transcriptomics has been combined with ATAC‐seq and scRNA‐seq, providing information about the space and time of gene expression in mouse fibroblasts during the wound healing process.^8^ Other technologies facilitating the incorporation of multiple‐omic approaches, include single‐cell triple‐omics sequencing (scTrio‐seq),^93^ which combines genetic, epigenetic and transcriptomic profiling, as well as genome and transcriptome sequencing (G&T‐seq).^94^ These technologies provide high‐throughput omic readouts that could be applied to molecular medicine and dermatology research.

Going forward, with a multitude of ‐omics modalities and computational frameworks for integration being introduced on a regular basis, different areas of dermatological science stand to benefit from the combination of particular technologies. For example, the analysis of inflammatory skin conditions via scRNA‐seq in conjunction with T‐cell and B‐cell sequencing and focused immunoproteomics, either with a method like CITE‐seq or SCoPE‐MS, could provide deeper insights into immune cell population activation state and reveal disease‐associated cell subtypes. On the other hand, in skin cancers, the combination of scRNA‐seq with spatial transcriptome sequencing could be advantageous in unravelling localized gene expression differences between the tumor microenvironment, tumor‐adjacent tissue and healthy tissues, as well as in mapping the trajectory and cross‐talk between malignant and non‐malignant cells at distinct stages of cancer development, invasion and metastasis. As single‐cell technologies evolve even further, we anticipate gaining an increasingly better understanding of the pathobiology of certain skin diseases and identifying key cell populations that play important roles in disease progression. Such progress offers scope to target therapeutically biologic pathways with new or repurposed drugs and develop better treatments for skin diseases.

## CONFLICTS OF INTEREST

The authors declare no conflicts of interest.

## **AUTHOR** **CONTRIBUTIONS**

G.T. and A.O. involved in conceptualization. A.O., S.T. and G.T. involved in investigation and writing—original draft. A.O. involved in supervision. G.T., A.V., J.A.M. and A.O. involved in writing—review & editing. S.T. involved in visualization.
